# Vertically-aligned BCN Nanotube Arrays with Superior Performance in Electrochemical capacitors

**DOI:** 10.1038/srep06083

**Published:** 2014-08-15

**Authors:** Junshuang Zhou, Na Li, Faming Gao, Yufeng Zhao, Li Hou, Ziming Xu

**Affiliations:** 1 Key Laboratory of Applied Chemistry, Department of Applied Chemistry, Yanshan University, Qinhuangdao 066004, P. R. China; 2 These authors contributed equally to this work.

## Abstract

Electrochemical capacitors (EC) have received tremendous interest due to their high potential to satisfy the urgent demand in many advanced applications. The development of new electrode materials is considered to be the most promising approach to enhance the EC performance substantially. Herein, we present a high-capacity capacitor material based on vertically-aligned BC_2_N nanotube arrays (VA-BC_2_NNTAs) synthesized by low temperature solvothermal route. The obtained VA-BC_2_NNTAs display the good aligned nonbuckled tubular structure, which could indeed advantageously enhance capacitor performance. VA-BC_2_NNTAs exhibit an extremely high specific capacitance, 547 Fg^−1^, which is about 2–6 times larger than that of the presently available carbon-based materials. Meanwhile, VA-BC_2_NNTAs maintain an excellent rate capability and high durability. All these characteristics endow VA-BC_2_NNTAs an alternative promising candidate for an efficient electrode material for electrochemical capacitors (EC).

Much more consideration of energy conservation and environment protection brings about urgent demands on clean, sustainable and renewable resources. However, most of the renewable energy (e.g. solar and wind power) is intermittent and cannot meet the needs for casual use. This situation demands energy storage systems to store the electricity generated from renewable sources. Batteries and electrochemical capacitors emerge at this moment^1^. However, future systems ranging from portable systems to automotive applications and large industrial equipments need a storage device with the capability to obtain higher energy and power density^2^. EC have attracted great attention very recently. They have a higher power density, quicker charge/discharge rate, and longer life-cycle compared with batteries^3^,^4^,^5^. EC are expected to be an important candidate in complementing or replacing batteries in the energy storage field. Consequently, many governments and enterprises have invested tremendous amounts of time and money into exploring, researching, and developing EC technologies^6^.

To meet the higher requirements of future systems, the performance of EC should be improved substantially. One of the most intensive approaches is the development of new electrode materials. In recent years, the huge progress in nanoscience and nanotechnology has provided an impetus for the development of new supercapacitor electrode structures. Many materials have been investigated as the electrode materials in supercapacitors, including transition metal oxides^7^,^8^, carbonaceous materials^9^,^10^ and conducting polymers^11^,^12^. Properties of electrode materials play an important role in determining the performances of the supercapacitors. Carbon materials, which possess stable physicochemical properties, high surface areas, good conductivity, and low cost^5^,^13^, have been commercially applied in EC for years. Even so, in spite of their large specific surface areas for charge storage, the EC performance cannot be enhanced illimitably. It is because the charges physically stored in porous electrode layers are limited^14^, and the electrical conductivity suffers from a decrease with increasing porosity^15^,^16^. Therefore, a new type of nanomaterial with good electrical conductivity and superior EC performance are highly desirable. The supercapacitor performance can also be enhanced by chemical functionalization of carbon materials with various heteroatoms, which could provide redox characteristics for improved pseudocapacitance. Recent studies have shown that N and B co-doping of carbon materials could enhance its specific capacitance due to a synergistic pseudocapacitive effect^17^,^18^. Herein, we report an unusually high capacitance (547 F/g at the current density of 0.2 Ag^−1^ in 6.0 M aqueous KOH electrolyte) for our newly developed vertically-aligned BC_2_N nanotube arrays (VA-BC_2_NNTAs) by simple low temperature solvothermal route without post-processing. The well-defined aligned pore channels combining favorable polar bond structure make VA-BC_2_NNTAs exhibit superior ability for EC technologies. The aligned morphology of VA-BC_2_NNTAs could effectively facilitate the transportation of electrolyte ions, and the nonbuckled tubular structure guarantees the large surface area to ensure abundant electrolyte ions transport during the charge/discharge process. Both the polar bond structure of VA-BC_2_NNTAs and the aligned nonbuckled hollow tube structure could contribute to the high specific capacitance for VA-BC_2_NNTAs. Compared with the nonaligned BC_2_N nanotubes (BC_2_NNTs) and vertically-aligned carbon nanotubes (VA-CNTs) with nonpolar bond, VA-BC_2_NNTAs show the highest specific capacitance with an excellent rate capability and high durability, and thus are attractive as electrode materials for EC applications.

Scanning electron microscopy (SEM) image of as-synthesized VA-BC_2_NNTAs was shown in Figure 1a. It clearly exhibits the highly ordered 3D array structures and a very good alignment of BC_2_N nanotube arrays with high packing densities. Meanwhile, SEM image of the resulting electrode (see Supplementary Fig. S1a online) reveals that the material in the final electrode is still aligned BC_2_N nanotubes, and the mean free path length of the ions (ca. 9.5 μm) is much longer than the distance between nanotubes. Selected area electron diffraction (SAED) pattern recorded from VA-BC_2_NNTAs confirms their high crystallinity (Figure 1b). The values of SAED pattern correspond to (002), (100), and (110) planes of BC_2_N structure, which are fairly consistent with the theoretical XRD pattern (JCPDS file no. 52–0233). High-resolution transmission electron microscopy (HRTEM) image of an individual BC_2_N nanotube is shown in Figure 1c. The lattice spacing is 0.323 nm, which is consistent with the (002) lattice planes of BC_2_N. Both HRTEM and SAED patterns confirm that the composition of the products is BC_2_N. The SEM images for the non-aligned BC_2_N nanotubes (BC_2_NNTs) and the corresponding resulting electrode can be found as Supplementary Fig. S2a, and Fig. S1b online.

TEM image of an individual BC_2_N nanotube (Figure 1e) reveals that the VA-BC_2_NNTAs have clean and smooth surfaces with the straight nonbuckled hollow tube structure, which is in contrast to the previously reported bamboo-like structures of BCN nanotubes^18^,^19^,^20^,^21^,^22^,^23^,^24^, but in line with the single-wall BCN nanotubes obtained via bias-assisted hot filament CVD route^25^. The straight hollow tube geometry was also confirmed by a top-view SEM image shown in Figure 1d, from which can be seen the straight nonbuckled tubular structure along the nanotube length. From the research results so far reported, most directly synthesized BCN nanotubes exhibit bamboo-like structure^18^,^19^,^20^,^21^,^22^,^23^,^24^. In particular interest, for the products obtained by our simple method, VA-BC_2_NNTAs display a characteristic feature of the smooth hollow cores without transverse layers shown in Figure 1d,e. This structure is noteworthy because it could indeed advantageously enhance capacitor performance (see below).

In order to study the distribution of B, C, and N species in VA-BC_2_NNTAs, the energy-dispersive X-ray (EDX) spectroscopy elemental mapping of VA-BC_2_NNTAs were carried out. Figure 1f–i shows a low-magnified SEM image of VA-BC_2_NNTAs, as well as elemental maps, representing B, C, and N, respectively. The elemental mapping of two single BC_2_N nanotubes (see Supplementary Fig. S3 online) reveals that B, C, and N species are homogeneously distributed in an individual BC_2_N nanotube, thus we can conclude that VA-BC_2_NNTAs possess a consistent B-C-N structure.

The presence of B, C, and N in the grown nanotubes was confirmed by EELS analysis of the K-edges of B, C, and N on the different parts of a single nanotube, shown in Figure 2. All EELS exhibit ionisation edges at ca. 191, 283 and 398 eV, corresponding to the K-shell of B, C and N, respectively^23^. Two characteristic peaks at 283 and 293 eV for C K-edge confirm the presence of graphitic carbon in the nanotube. These two peaks are attributed to a 1s-π* transition and a series of 1s-σ* transitions, respectively. The defined π* and σ* fine structure features of the C K-edge are signs of well-graphitized sp^2^-bonding carbon networks^26^. The B and N K-edge signals also show a discernible π* peak as well as a σ* band. It indicates that the B and N atoms are in the same sp^2^-hybridized state as their C counterparts^27^,^28^,^29^,^30^,^31^. The low-energy peak (191 eV) for B K-edge can be accounted for B bonded to C, and the high-energy peak (198 eV) originates from B bonded to N^32^. These results clearly revealed that the resultant nanotubes are made up of B, C, and N, and the EELS obtained from different parts of a single nanotube are almost the same, indicating VA-BC_2_NNTAs with the homogeneous distribution of B, C, and N species.

The chemical composition of VA-BC_2_NNTAs was determined quantitatively from EELS. For EELS elemental quantification, among the chemically bonded C, B, and N atoms, the C content of VA-BC_2_NNTAs (55%) dominates over B (19%) and N (26%), the overall stoichiometry reveals the local chemical composition, B_19_C_55_N_26_, of the ternary BCN compound. The ratio of B, C and N is approximately constant with the ratio of 1:2:1, which is close to BC_2_N. Recent studies have showed that N and B co-doping of porous carbon could enhance its specific capacitance due to a synergistic pseudocapacitive effect^17^,^18^. Generally, Nitrogen atoms can easily be introduced into the carbon layer, whereas the synthesis of B-doped carbons is much more difficult than the case of N-doped carbons^33^. The previous reports on the B-doping effect contain a very small amount of boron^17^,^34^,^35^. In contrast, our obtained BCN nanotubes contains a larger amount of boron in the carbon layer (B/C is ca.0.345), thereby making the synergetic effect of N and B co-doping more remarkable. It may be another reason why our obtained BCN nanotubes have the higher capacitance.

The ternary bonding nature of the VA-BC_2_NNTAs was further confirmed by X-ray photoelectron spectroscopy (XPS) characterization. Figure 3a shows XPS survey spectrum of VA-BC_2_NNTAs. The presence of an O 1s peak around 532 eV in VA-BC_2_NNTAs is possibly due to the incorporation of physicochemically adsorbed oxygen^35^,^36^. The high-resolution B 1s XPS spectrum given in Figure 3b could be deconvoluted into mainly two subpeaks at 189.6 and 191.5 eV, arising from the B-C and B-N bond, respectively. The relatively higher intensity of the B-C peak than that of B-N indicates that a greater number of B is attached to C in the network. The predominant asymmetric C 1s peak shown in Figure 3c indicates the existence of C-N or C-B bonds in the graphitic network. The four deconvoluted peaks in the C 1s spectrum at 283.6, 284.6, 286.2, and 288.5 eV could be assigned to C-B, C-C, C-N, and C-O bonds, respectively. The high-resolution N 1s XPS spectrum in Figure 3d has been fitted with three subpeaks at 397.4, 398.5, and 399.8 eV, attributable to the N-B bond, graphitic N-C bond, and pyridinic N-C bond, respectively. The amount of pyridinic N is relatively smaller than the graphitic nitrogen. From the XPS spectra of B 1s and N 1s core-level electrons, the presence of sp^2^ B-C, C-N, and B-N bonding states can be clearly identified.

To evaluate the properties of VA-BC_2_NNTAs as EC electrodes, cyclic voltammetry (CV) was used in determination of electrochemical properties of the samples. Figure 4a compares CV curves of VA-CNTs, BC_2_NNTs, and VA-BC_2_NNTAs electrodes for a three-electrode cell at a scan rate of 5 mV/s. The TEM image of VA-CNTs can be found as Supplementary Fig. S2b online. From the CV curves shown in Figure 4a, the remarkable differences in CV curve shape between VA-CNTs, BC_2_NNTs, and VA-BC_2_NNTAs can be easily recognized. In detail, VA-CNTs exhibited small rectangular curve corresponding to a low capacitance, while BC_2_NNTs presents capacitive behavior with the appearances of a larger rectangular-like shape in CV curve. From the bigger CV loop observed for BC_2_NNTs than that of the VA-CNTs, it indicates a thicker double-layer region for BC_2_NNTs electrode. This phenomenon may be due to the heteropolar B-N bonding, which could induce an extra dipole moment^37^ and may enhance the wettability between the electrolyte and electrode materials, thus could improve the electric double-layer capacitance. Compared with VA-CNTs and BC_2_NNTs, VA-BC_2_NNTAs present the best capacitive performance with a largest rectangular-like shape and clear humps of the voltammetry characteristics. It implies that the aligned nonbuckled hollow tube structure can contribute to the high specific capacitance for VA-BC_2_NNTAs.

It is worth noting that the electrochemical behavior of randomly entangled BC_2_NNTs electrode shows a rather limited capacitance compared to VA-BC_2_NNTAs. It is most probably because the randomly entangled BC_2_NNTs are unable to support a facilitated access of the electrolyte ions due to the mismatch between the irregular pore structures. (see Figure 4b) Unlike BC_2_NNTs, superior electrochemical properties of VA-BC_2_NNTAs originate from better ion diffusivity of VA-BC_2_NNTAs steming from the aligned pore structures compared with BC_2_NNTs, hence showing a much higher capacitance compared with BC_2_NNTs.

The galvanostatic charge/discharge measurement is considered to be a more accurate technique especially for pseudocapacitances^38^. Therefore, galvanostatic charge/discharge experiments are performed with various current densities between −0.8 and 0.2 V in order to further investigate the performances of all samples. Figure 4c shows the V–t plots of all samples at the constant current of 0.2 Ag^−1^. The specific capacitance of the VA-BC_2_NNTAs electrode in 6 M KOH is 547 F/g, which is significantly higher than that of BC_2_NNTs (70.18 F/g) and VA-CNTs (41 F/g). VA-BC_2_NNTAs display the highest specific capacitance. We attribute this excellent capacitive performance to the facile ion transport in the open aligned structure. From Supplementary Fig. S1a online, it can be seen that the mean free path length of the ions is much longer than the distance between BCN nanotubes. The aligned morphology of VA-BC_2_NNTAs could effectively facilitate the transportation of electrolyte ions during the charge/discharge process, thus improve the capacitance.

Moreover, we compared our results with major published data on carbon-based materials (see Table S1) and listed the major characteristics of each report, such as the origin, used electrolytes, the specific capacitances (*Cs*) and the cycling stability. The *Cs* values of these carbon-based materials ranged from 80 F/g to 385 F/g. Clearly, our *Cs* value of VA-BC_2_NNTAs, 547 F/g is much higher than any of the involved carbon-based materials, and also much larger than that of the aligned BCN nanoatubes with bamboo-like structure (312.0 F/g)^18^. It indicated that in spite of the aligned structure inherent in VA-BC_2_NNTAs, the unique nonbuckled tubular structure could be another key factor for the contribution of the high *Cs*. This is because the nonbuckled tubular morphology of our obtained BC_2_N nanotubes could effectively facilitate the transportation of electrolyte ions during the charge/discharge process. For the BCN nanoatubes with bamboo-like structure, the transverse layers possess higher ion diffusion barriers in the inner region of the electrode, resulting in higher internal resistance and inferior capacitance performance. Consequently, this structure inherent in VA-BC_2_NNTAs can dramatically enhance the specific capacitance and VA-BC_2_NNTAs are attractive to be used as electrode materials for EC applications.

Figure 4d represents the relationships between specific capacitance and charge/discharge current density to study the rate capability of the electrode materials. The capacitance retention is defined as the ratio of the specific capacitance at various current densities to that at 0.2 A/g. The capacitance retention of VA-BC_2_NNTAs retains 84% as current density increases from 0.2 to 2 A/g, which is significantly higher than that of the BC_2_NNTs (68%) and VA-CNTs (66%). Obviously, VA-BC_2_NNTAs are demonstrated to have very high rate-capability. The unique structural feature has remarkably improved the capacitance performance of VA-BC_2_NNTAs electrode at high charge/discharge rate, and this is very important for the applications where a high rate of discharge-recharge is required. Long cycling life is another important requirement for EC. The cycling life test was carried out by repeating the charge/discharge test at a current density of 1 A/g for the first 1500 cycles, and 5 A/g for the last 2000 cycles. As can be seen from Figure 4e, the VA-BC_2_NNTAs electrode exhibits an excellent electrochemical stability with only 3% deterioration after 3500 cycles.

In summary, our studies have outlined a general and rational strategy to fabricate the high-densely packed VA-BC_2_NNTAs by simple low temperature solvothermal route. Such VA-BC_2_NNTAs possess an ingenious structure with vertically-aligned morphology containing no transverse layers which endow an unusually high capacitance. These studies represent substantial progress towards high capacitance, excellent rate capability, and outstanding cycling stability produced by VA-BC_2_NNTAs, opening the possibility to engineer capacitor electrodes based on VA-BC_2_NNTAs in order to target a wide range of applications. These advances may extend the frontier of EC research and open up new paths to accelerate development of EC applications.

## Experimental Section

### Materials synthesis

The vertically-aligned BC_2_N nanotube arrays (VA-BC_2_NNTAs) were synthesized as follow. In our experiments, methyl cyanide (CH_3_CN) was distilled at 82°C to remove the impurities and moisture. The other reagents were analytically pure and used without further purification. The solvothermal reaction was carried out in a stainless steel autoclave (40 mL in total capacity) under autogenous pressure. All the manipulations were carried out in a dry glove box with flowing N_2_. In the typical process, 2.5 g sodium azide (NaN_3_), 2.5 g ammonium fluoroborate (NH_4_BF_4_) and 0.5 g hexadecyl trimethyl ammonium bromide (CTAB) were put into a stainless steel autoclave, and then the autoclave was filled with 4 mL anhydrous CH_3_CN and 24 mL benzene. The autoclave was sealed and maintained at 400°C for 14 h in a furnace, then it was allowed to cool to room temperature naturally. The products were collected and washed with distilled water, absolute ethanol and hydrochloric acid several times to remove the impurities. Then the final product was dried in vacuum at 65°C for 8 h. For comparison purposes, nonaligned BC_2_N nanotubes (BC_2_NNTs) were grown under similar conditions except for CTAB. VA-CNTs was purchased from Beijing DK nano technology Co.LTD, and used without purification.

### Characterization

The morphology of the nanotubes was analyzed by scanning electron microscopy (SEM, Hitachi S-4800) and transmission electron microscopy (TEM, Hitachi H-7650). A few powder samples were placed onto silver glue, which was adhered to the SEM stainless steel sample holder. TEM samples were prepared by placing a droplet (20 μL) of our sample onto a 3 mm carbon-coated copper grid for 5 min. Afterwards, the excess water evaporated at room temperature. The TEM investigations were operated at 120 kV for imaging. High–resolution transmission electron microscopy (HRTEM) and selected-area electron diffraction (SAED) were used to investigate the phase structure of sample by TEM using a JEM-2010 transmission electron microscope. The electron energy-loss spectroscopy (EELS) and energy dispersive x-ray spectroscopy (EDX) based elemental mapping were used to determine the chemical composition of the sample. X-ray photoelectron spectroscopic (XPS) measurements were performed on a ESCALAB 250 X-ray Photon-electron Spectroscopy.

### Electrochemical measurements

Electrodes were fabricated by mixing 80 wt% VA-BC_2_NNTAs, similarly other active materials (i.e., VA-CNTs or BC_2_NNTs), 10 wt% acetylene black, and 10 wt% poly tetra fluoro ethylene (PTFE) binder. The loading amount of all materials in final electrodes is 2.6 mg. The mixture was mixed with absolute ethyl alcohol and heated at 60°C in water bath to form slurries. The homogenous slurries were coated onto nickel mesh (1 cm^2^ area) and further dried at 120°C for 12 h under vacuum. As-formed electrodes were then pressed at a pressure of 4 MPa.

Cyclic voltammetric (CV) studies were performed by CHI 832C electrochemical workstation (Shanghai Chenhua, China) in the potential range of −0.8 ~ 0 V vs Hg/HgO at the scan rate of 5 mVs^−1^. Galvanostatic charge/discharge cycles were measured by a Land cell tester (CT2001A) at 0.2 ~ 2 Ag^−1^ over a voltage range of −0.8 ~ 0.2 V vs Hg/HgO. The electrochemical cell used here was three-electrode cell filled with the electrolyte of 6.0 M KOH aqueous solution. A standard three–electrode cell was employed with an Hg/HgO electrode as reference electrode, a platinum plate as counter electrode, and the active material composite (i.e., VA-CNTs, BC_2_NNTs or VA-BC_2_NNTAs) was used as working electrode. The specific capacitance (*Cs*) of the systems was calculated according to the following equation: 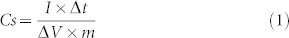
where *Cs* (Fg^−1^) is the specific capacitance, *I* (A) refers to the discharge current, Δ*V* (V) represents the potential change within the discharge time Δ*t* (s), and *m* (g) corresponds to the amount of active material on the electrode. All the experiments were conducted at room temperature (25 ± 1°C).

## Author Contributions

J.Z., N.L. and F.G. wrote the main manuscript text and Y.Z., L.H. and Z.X. prepared figures. All authors reviewed the manuscript.

## Supplementary Material

Supplementary Information

## Figures and Tables

**Figure 1:**
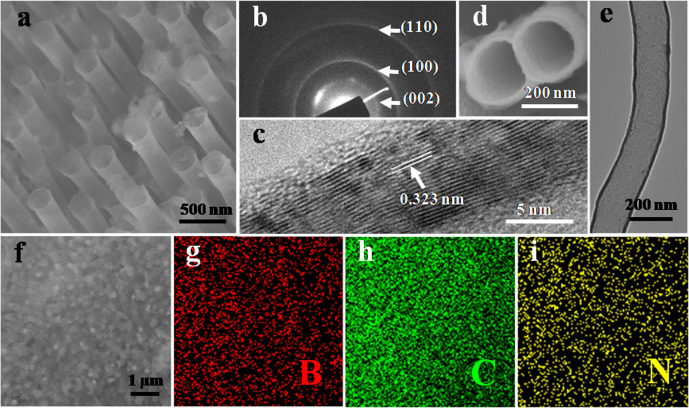
(a) High-magnified SEM image of VA-BC_2_NNTAs, (b) SAED pattern and (c) HRTEM image of VA-BC_2_NNTAs, (d) Top-view SEM image and (e) TEM image of VA-BC_2_NNTAs, (f) Low-magnified SEM image of VA-BC_2_NNTAs, (g–i) The corresponding EDX mapping of (g) boron (red), (h) carbon (green), and (i) nitrogen (yellow) from SEM image (f). All samples are as-synthesized VA-BC2NNTAs rather than the final electrode.

**Figure 2:**
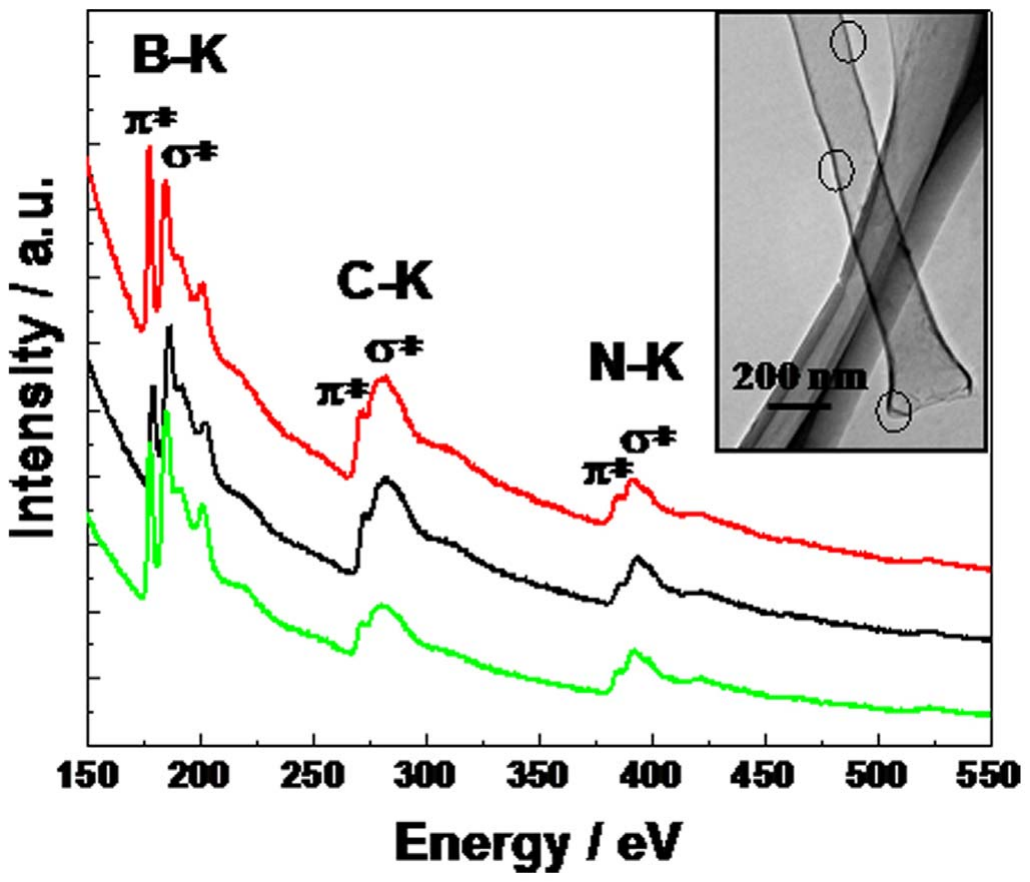
EELS spectra obtained from different parts of an individual vertically-aligned BC_2_N nanotube as shown in circles in TEM image (inset).

**Figure 3:**
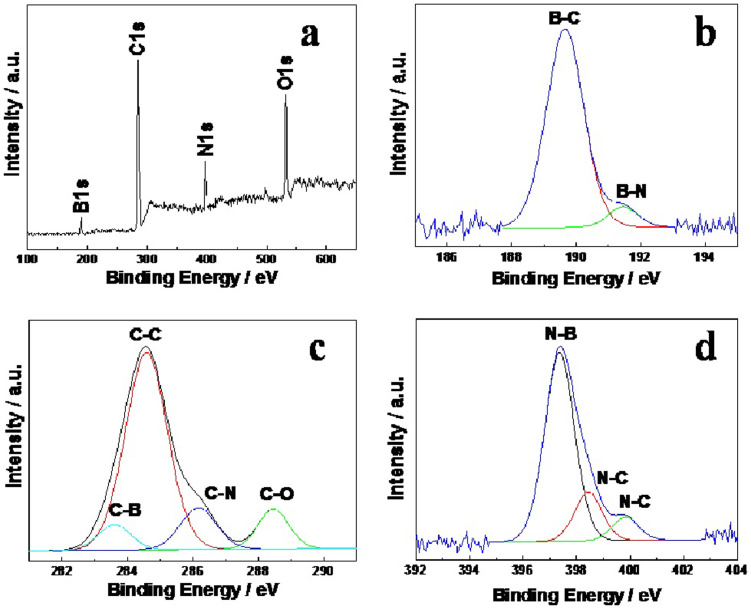
(a) XPS survey spectrum of VA-BC_2_NNTAs. (b–d) High-resolution XPS spectra of B 1s, C 1s, and N 1s of VA-BC_2_NNTAs, respectively.

**Figure 4:**
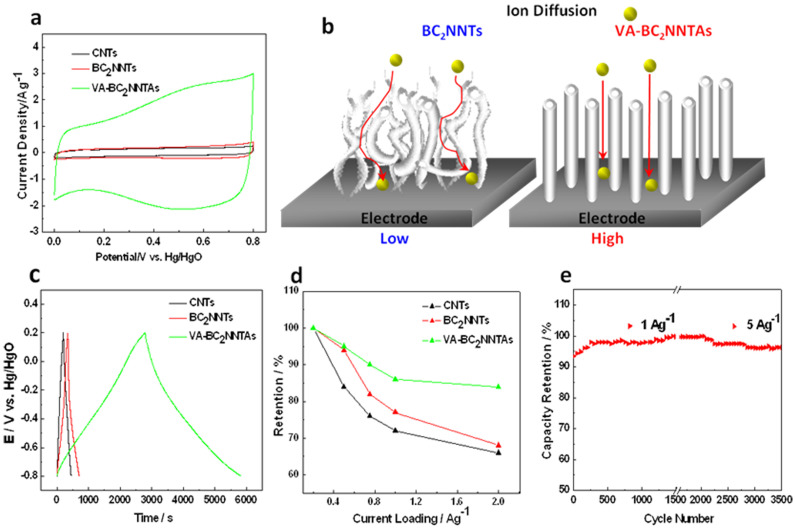
(a) CV curves of VA-CNTs, BC_2_NNTs, and VA-BC_2_NNTAs samples in 6 M KOH solution at a scan rate of 5 mV/s, (b) Schematic model comparing the ion diffusion for BC_2_NNTs and VA-BC_2_NNTAs, (c) charge/discharge curves of VA-CNTs, BC_2_NNTs, and VA-BC_2_NNTAs samples in 6 M KOH solution at a current density of 0.2 A/g, (d) corresponding capacity retentions at the current density from 0.2 to 2 A/g, (e) stability evaluation of the VA-BC_2_NNTAs electrode material in 6 M KOH solution at a charge current of 1 A/g for the first 1500 cycles, and 5 A/g for the last 2000 cycles.
