# Supplementary material for: Vertically-aligned BCN Nanotube Arrays with Superior Performance in Electrochemical capacitors
Source: Sci Rep. 2014 Aug 15;4:6083. doi: 10.1038/srep06083 (PMC4133702; doi:10.1038/srep06083)
Supplement: Supplementary Information [file srep06083-s1.pdf]

Supporting Information

Vertically-aligned BCN Nanotube Arrays with Superior Performance in Electrochemical capacitors

Junshuang Zhou,† Na Li,† Faming Gao*, Yufeng Zhao, Li Hou and Ziming Xu

Key Laboratory of Applied Chemistry, Department of Applied Chemistry, Yanshan University, Qinhuangdao 066004, P. R. China.

*E-mail: fmgao@ysu.edu.cn. Phone: 86 335 8387552. Fax: 86 335 8061569

†J.Z. and N.L. contributed equally.

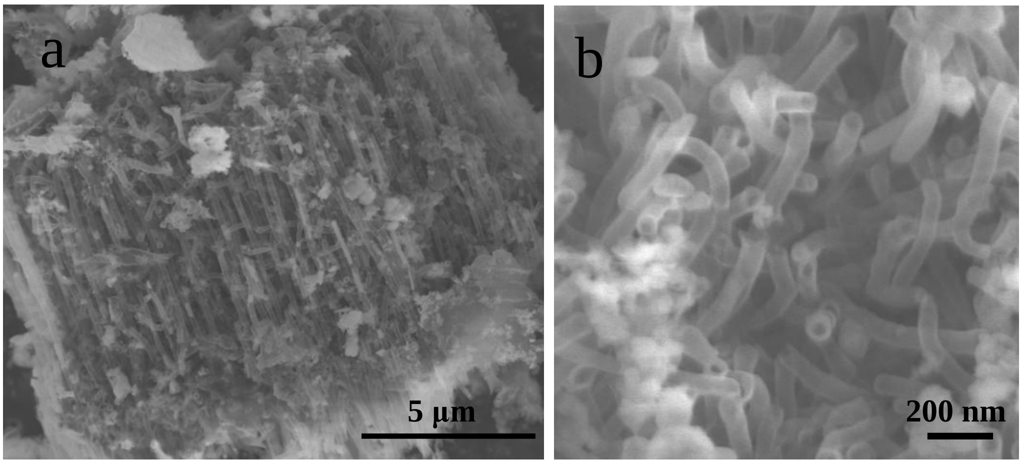

Figure S1. The SEM images of the final electrode (a) VA-BC_2NNTAs (b) BC_2NNTs

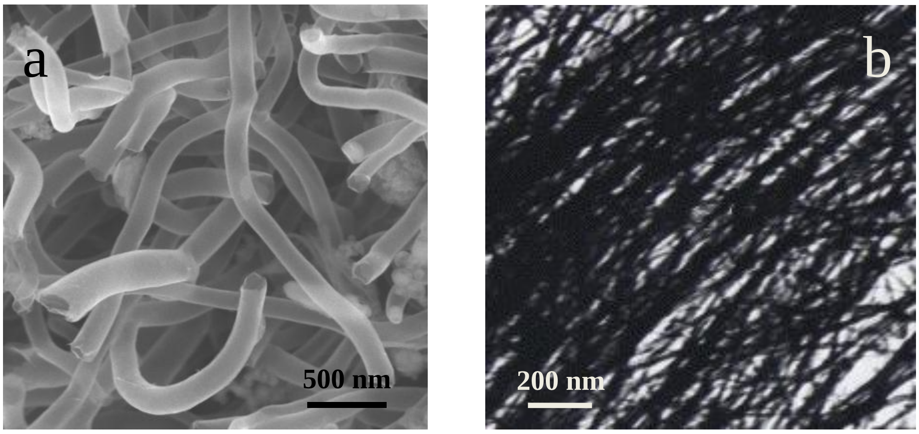

Figure S2. (a) SEM image of BC_2NNTs; (b) TEM image of VA-CNTs.

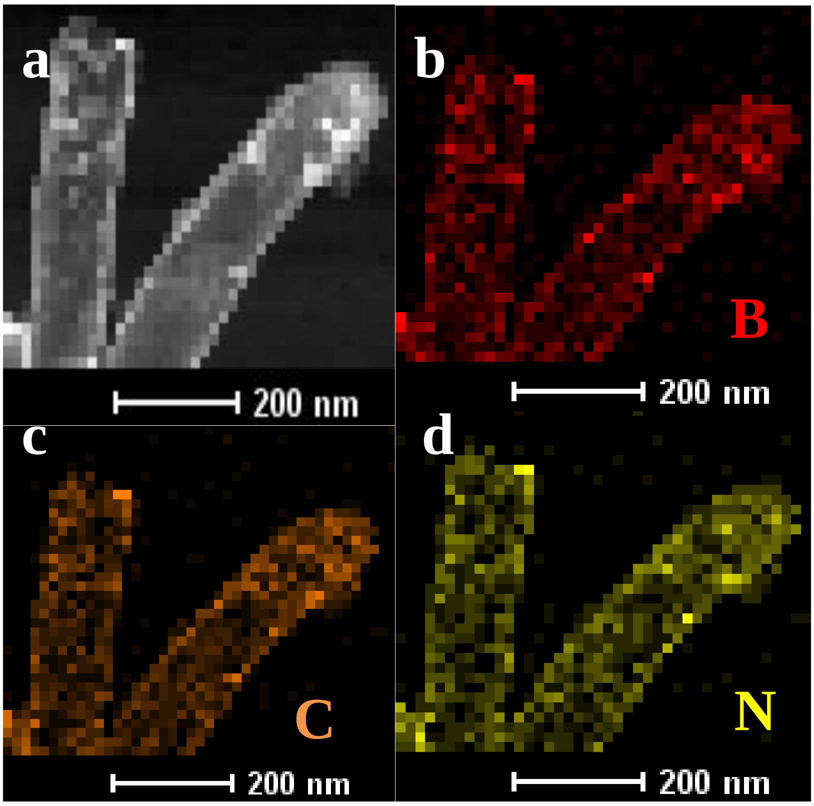

Figure S3. HAADF-STEM image (a) and STEM-EDX maps (b-d) of two single BC_2N nanotubes

Table S1. Comparison of the *Cs* and cycling stability of VA-BC_2NNTAs and Some Carbon-based Materials in the Literatures

Item	Journal (Year)	Voltage window (V)	Electrolyte	Loading data (mg/cm^2)	*Cs* (F/g)	Cycling Stability	Ref
activated microwave exfoliated graphite oxide (a-MEGO)	Science (2011)	3.5	BMIM BF_4/AN	2.5~4	166	with 3% deterioration after 10000 cycles	1
Single-walled carbon nanotubes	Nat. Mater. (2006)	2.5	1 M Et_4NBF_4	No data	80	No data	2
vertically aligned BCN nanotubes	ACS Nano (2012)	1.3	6 M KOH	No data	321	with 5% deterioration after 1000 cycles	3
3D N and B co-doped graphene	Adv. Mater. (2012)	1.0	1 M H_2SO_4	No data	239	without degradation after 1000 cycles	4
B and N co-doped porous carbon	J. Power Sources (2009)	0.8	6 M KOH	No data	268	No data	5
3D-CNT/graphene sandwich	Adv. Mater. (2010)	0.65	6 M KOH	No data	385	increase ca. 20% after 2000 cycles	6
N-containing hydrothermal carbons	Adv. Mater. (2010)	1.0	6 M KOH	No data	220	with 5% deterioration after 2000 cycles	7
carbonaceous materials derived from fungi (P-HT-A)	Adv. Mater. (2011)	1.4	6 M KOH	No data	196	with 8.16% deterioration after 1000 cycles	8
carbonized eggshell membrane (CESM)	Adv. Energy Mater. (2012)	1.0	1 M KOH	No data	297	with 3% deterioration after 10000 cycles	9
porous graphite particles	Adv. Energy Mater. (2011)	2.7	1 M Et_4NBF_4	4~12	102	with 2% deterioration after 5000 cycles	10
graphene/MnO_2/CNT composite film	Nano Lett. (2012)	1	1 M Na_2SO_4	No data	372	with 5% deterioration after 1000 cycles	11
N-doped porous carbon nanofibers	ACS Nano (2012)	1	6 M KOH	5~6	202	with 3% deterioration after 3000 cycles	12
this work	-	1	6 M KOH	2.6	547	with 3% deterioration after 3500 cycles [a]	-

[a] The cycling life test was carried out by repeating the charge/discharge test at a current density of 1 A/g for the first 1500 cycles, and 5 A/g for the last 2000 cycles.

Reference

[1] Zhu, Y. Murali, S. Stoller, M. D. Ganesh, K. J. Cai, W. Ferreira, P. J. Pirkle, A. Wallace, R. M. Cychosz, K. A. Thommes, M. Su, D. Stach, E. A. Ruoff, R. S. *Science* **2011**, *332*, 1537.

[2] Futaba, D. N. Hata, K. Yamada, T. Hiraoka, T. Hayamizu, Y. Kakudate, Y. Tanaike, O. Hatori, H.; Yumura, M.; Iijima, S. *Nat Mater* **2006**, *5*, 987.

[3] Iyyamperumal, E. Wang, S. Dai, L. *ACS Nano* **2012**, *6*, 5259.

[4] Wu, Z.-S. Winter, A. Chen, L. Sun, Y. Turchanin, A. Feng, X. Müllen, K. *Advanced Materials* **2012**, *24*, 5130.

[5] Guo, H. Gao, Q. *Journal of Power Sources* **2009**, *186*, 551.

[6] Fan, Z. Yan, J. Zhi, L. Zhang, Q. Wei, T. Feng, J. Zhang, M. Qian, W. Wei, F. *Advanced Materials* **2010**, *22*, 3723.

[7] Zhao, L. Fan, L.-Z. Zhou, M.-Q. Guan, H. Qiao, S. Antonietti, M. Titirici, M.-M. *Advanced Materials* **2010**, *22*, 5202.

[8] Zhu, H. Wang, X. Yang, F. Yang, X. *Advanced Materials* **2011**, *23*, 2745.

[9] Li, Z. Zhang, L. Amirkhiz, B. S. Tan, X. Xu, Z. Wang, H. Olsen, B. C. Holt, C. M. B. Mitlin, D. *Advanced Energy Materials* **2012**, *2*, 431.

[10] Chen, Z. Wen, J. Yan, C. Rice, L. Sohn, H. Shen, M. Cai, M. Dunn, B. Lu, Y. *Advanced Energy Materials* **2011**, *1*, 551.

[11] Cheng, Y. Lu, S. Zhang, H. Varanasi, C. V. Liu, J. *Nano Letters* **2012**, *12*, 4206.

[12] Chen, L.-F. Zhang, X.-D. Liang, H.-W. Kong, M. Guan, Q.-F. Chen, P. Wu, Z.-Y. Yu, S.-H. *ACS Nano* **2012**, *6*, 7092.
